# Embryonic Stem Cell-Derived Exosomes Attenuate Transverse Aortic Constriction Induced Heart Failure by Increasing Angiogenesis

**DOI:** 10.3389/fcvm.2021.638771

**Published:** 2021-06-28

**Authors:** Yanan Pang, Minglu Ma, Dong Wang, Jiacun Xia, Xinyue Wang, Lei Hou, Zhiguo Wang, Xun Li

**Affiliations:** ^1^Department of Cardiology, The First Affiliated Hospital of Soochow University, Suzhou, China; ^2^Division of Cardiology, Tongren Hospital, Shanghai Jiao Tong University School of Medicine, Shanghai, China; ^3^Division of Hospital of Traditional Chinese Medicine, Qingdao Hiser Hospital, Qingdao, China

**Keywords:** embryonic stem cells, exosomes, angiogenesis, transverse aortic constriction, heart failure

## Abstract

**Background:** Although there are concerns regarding their clinical use, embryonic stem cells (ESCs) hold a great promise for cardiac repair. Exosomes deriving from ESCs constitute a promising alternative for heart restoration. However, their effects in hypertension-induced heart failure are still unknown.

**Objective and Methods:** To investigate the effects of ESCs-derived exosomes on hypertension-induced heart failure and the underlying mechanisms, sustained transverse aortic constriction (TAC) was performed on 8-week-old C57BL/6 male mice. After 1 months, ESCs-derived exosomes were isolated and injected intravenously once a week for 6 weeks. Echocardiography, wheat germ agglutinin (WGA), Masson staining, immunohistochemistry, and tube formation assays were all involved in our study.

**Results:** Proteomics analyses revealed that ESC-derived exosomes contain FGF2 protein. Tube formation induced by these exosomes could be inhibited by FGF2R siRNA interference. ESCs-derived exosomes evidently attenuated TAC-induced heart failure, improving cardiac function and promoting myocardial angiogenesis which can be attenuated by selective FGF2 inhibitor AZD4547.

**Conclusions:** ESC-derived exosomes attenuate TAC-induced heart failure mostly by promoting myocardial angiogenesis. FGF2 signaling plays a vital role in the myocardial angiogenesis induced by ESC-derived exosomes.

## Introduction

Compensatory adaptation occurs early in response to high blood pressure (1). However, persistent high blood pressure results in cardiac remodeling, which eventually leads to heart failure (2, 3). Inadequate blood supply accelerates the transition from compensatory cardiac hypertrophy to heart failure (4, 5). Adequate myocardial angiogenesis is important to maintain myocardial function in response to sustained hypertension. Previous studies have demonstrated the potential of embryonic stem cells (ESCs) in rescuing injured hearts, which is due to their considerable differentiation ability (6–8). However, ESCs pose some challenges for clinical use with respect to immune tolerance and cell retention. ESC-derived exosomes, which carry donor-specific microRNAs and proteins, may be a promising alternative for heart failure treatment (9, 10).

In this study, we found that systemic administration of ESC-derived exosomes attenuated transverse aortic constriction (TAC)-induced heart failure by promoting myocardial angiogenesis. Furthermore, we found that fibroblast growth factor-2 (FGF2) signaling played a vital role in this process.

## Materials and Methods

### Cell Culture

We cultured human umbilical vein endothelial cells (HUVEC) and human embryonic stem cells (ESC) in our laboratory for this experiment. Our culture protocols for these two cells strictly followed the description of Chen et al. (10).

### Isolation and Identification of Exosomes From ESCs

The culture medium of the ESCs was collected, and exosomes were isolated using the methods described previously (11). After ultracentrifugation, the exosomes were fixed in the fixative and their morphology was observed by transmission electron microscope (TEM; Hitachi H-7650). The size distribution and particle concentration of exosomes were measured using the qNano platform (iZON® Science, UK). Expression of the exosomal markers CD9 (1:1,000; Epitomics) and Alix (1:1,000; Epitomics) was analyzed using Western blotting.

### Proteomic Analysis of ESC-Derived Exosomes

ESC-derived exosomes were lysed in 8 M urea and 100 mM Tris solution (pH 7.6). After reduction by dithiothreitol and alkylation by iodoacetamide, the protein solution was digested by trypsin at 37°C for 18 h. Then, the peptide solution was transferred to a solid phase extraction cartridge for desalting and clean-up of the sample. The samples were analyzed with a QExactive HF mass spectrometer (Thermo Fisher Scientific, San Jose, CA, USA) equipped with a Nanospray Flex source (Thermo Fisher Scientific). Each sample was separated by an in-house micro-tip C18 column (75 × 200 mm) packed with ReproSil-Pur C18-AQ 3.0-mm resin (Dr. Maisch GmbH, Germany) on an Easy-nLC 1200 nanoflow HPLC system (Thermo Fisher Scientific). The MS1 full scan was performed at a resolution of 60,000 @ m/z 200, followed by “top 15” MS2 scans generated by HCD fragmentation at a resolution of 15,000 @ m/z 200. The normalized collision energy (NCE) was set at 28%, and the dynamic exclusion time was 45 s. Mass spectrometric data were analyzed using MaxQuant 1.6 against the human UniProt database containing 172,418 sequences. Carbamidomethyl cysteine was searched as a fixed modification. Oxidized methionine and protein N-term acetylation were set as variable modifications. Enzyme specificity was set to trypsin. The maximum number of missing cleavage sites was set to 2. The tolerances of the first search and main search for peptides were set to 20 and 4.5 ppm, respectively. The minimum peptide length was set to 7. The false discovery rates (FDRs) of peptides, proteins, and sites were all set to <0.01.

### FGF2R siRNA Transfection

The transfection operation was carried out according to the instructions of siRNA kit was purchased from OBIO Biotechnology Co. Ltd. Shanghai, China. Before transfection, inoculate 2^*^10^5^ HUVEC in each well of the 24-well plate and add 400 ul anti-free medium. At the time of transfection, the cell density reaches 30–50%. Dilute siRNA with 50 ul Opti-MEM to a final transfection concentration of 50 nM. After mixing, let it stand for 5 min. Subsequently, the volume (ul) of added PEI was three times the mass (ng) of siRNA. After violent shaking, let it stand again for 15–20 min. Add the transfection mixture to non-antibiotic medium and place it in a 37°C cell incubator. After 4–6 h, change to complete medium and incubate for 48 h.

### Endothelial Cell Culture and Tube Formation Assay

Matrigel (50 μl; BD Biosciences) was added to every well of a 96-well plate on ice and solidified at 37°C. HUVECs (3 × 10^4^ cells/100 μl) were mixed and cultured on solidified Matrigel plugs in DMEM at 37°C in humidified air with 5% CO_2_ for 6 h. Exosomes (1^*^10^9^) was added to medium as treated groups. The dosage and usage of siRNA were completely in accordance with the instructions of siRNA. Tube structures were counted in 3 randomly selected fields at 10× magnification.

### Animal Study

Totally, thirty-six 8-week-old C57BL/6 male mice were used in this study. TAC was performed on thirty mice as previously described (12). One month later, exosomes (3^*^10^10^) were injected into the tail vein of experimental mice three times a week for 6 weeks as exosome group (*n* = 10). AZD4547 (2 mg/kg/day) was administered via intra-peritoneal injection at the same time besides exosomes administration as TAC+exosome+AZD4547 group (*n* = 10). TAC mice received the same volume of PBS intravenously (*n* = 10) at the same timelines as exosome administration. Control mice did not receive TAC procedure after anesthesia (*n* = 6).

### Measurement of Cardiac Function

Echocardiographic images were obtained with a VisualSonics Vevo System (VisualSonics Inc., Canada) after 6 weeks treatment. The mice were anesthetized, and the heart rates were maintained between 450 and 500 beats per min. Both B- and M-mode images were acquired, and the left ventricular internal diastolic diameter (LVIDD), left ventricular interval systolic diameter (LVIDS), left ventricular ejection fraction (EF), and left ventricular fractional shortening (FS) were measured. All measurements were completed by two blinded experienced technicians. A total of 5–8 mice were analyzed per group.

### Histopathological Examination of Mouse Hearts

After the echo examination, the heart was harvested and the left ventricle was sliced from the apex to the base at 6-μm thickness for the evaluation of morphology and interstitial fibrosis. Sections were stained with Masson's trichrome. The percentage of LV fibrosis was determined using a previously described method (13). FITC-conjugated wheat germ agglutinin (WGA) was performed for further determination of cell size. Quantitative digital image analysis system (Image-Pro Plus 6.0) was used in image measurement. Capillary density was quantitatively measured microscopically at ×400 magnification for three randomly chosen fields. The ratio of CD31-positive cells to the total area was calculated by image analysis software. Samples from 4 to 6 mice per group were analyzed.

### Statistical Analysis

All values are expressed as the means ± standard errors. Comparisons between groups were performed using an unpaired *t* test. Two-way ANOVA was used to test for differences among groups. A *p* < 0.05 was considered statistically significant.

## Results

### Characterization of ESC-Derived Exosomes

**Figure 1:**
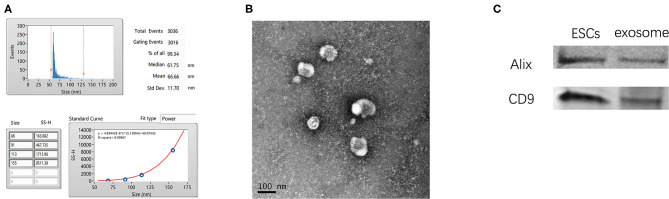
Characterization of ESC-derived exosomes. **(A)** Particle size distribution of ESCs-derived exosomes. **(B)** Morphology of ESCs-Exosomes observed by TEM (scale bar, 100 nm). **(C)** Western blotting showed the presence of exosome markers CD9 and Alix.

Exosomes were isolated from the cultured medium of ESCs. qNano analysis showed that most exosomes were in the range of 50–125 nm in size (Figure 1A). TEM imaging (Figure 1B) revealed exosomes with characteristic ball-shaped morphology. Additionally, vesicles from ESCs expressed the exosome marker proteins CD9 and Alix (Figure 1C).

### ESC-Derived Exosomes Contained Abundant FGF2

**Figure 2:**
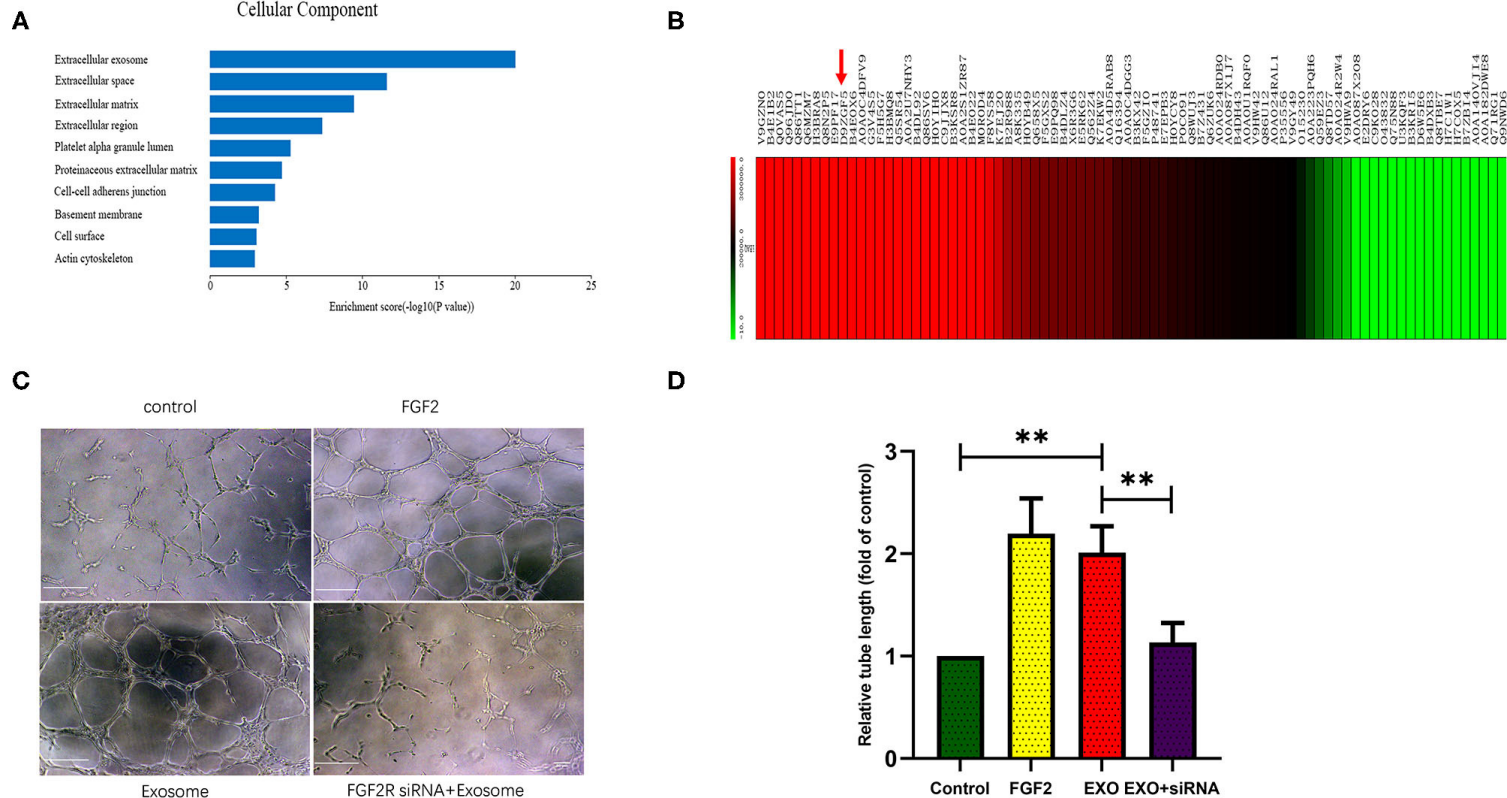
ESCs-derived exosomes stimulate angiogenesis in a FGF2 related way. **(A,B)** Proteome analysis showed ESCs-derived exosomes contains hundreds of proteins in which FGF2 was high abundant and ranked in the top ten of all the proteins in the exosomes. **(C,D)** Exosomes treated HUVECs formed greater tube length compared with control group. FGF2R siRNA interference impaired the exosomes induced angiogenesis *in vitro* (scale bar, 100 um). *p < 0.05, **p < 0.01.

Proteomic analysis of ESC-derived exosomes showed that EC-derived exosomes contained hundreds of proteins, among which FGF2 was highly abundant, ranking in the top ten of all proteins detected within exosomes (Figures 2A,B).

### The FGF2R siRNA Impaired Exosome-Induced Angiogenesis *in vitro*

Tube formation assays showed that exosome-treated HUVECs formed greater tube length than the control HUVECs (*p* < 0.01), as FGF2 treated HUVECS. The FGFR2 siRNA impaired exosome-induced tube formation (*p* < 0.01) (Figures 2C,D).

### ESC-Derived Exosomes Rescued Heart Failure After TAC Which Can Be Attenuated by AZD4547 Administration

**Figure 3:**
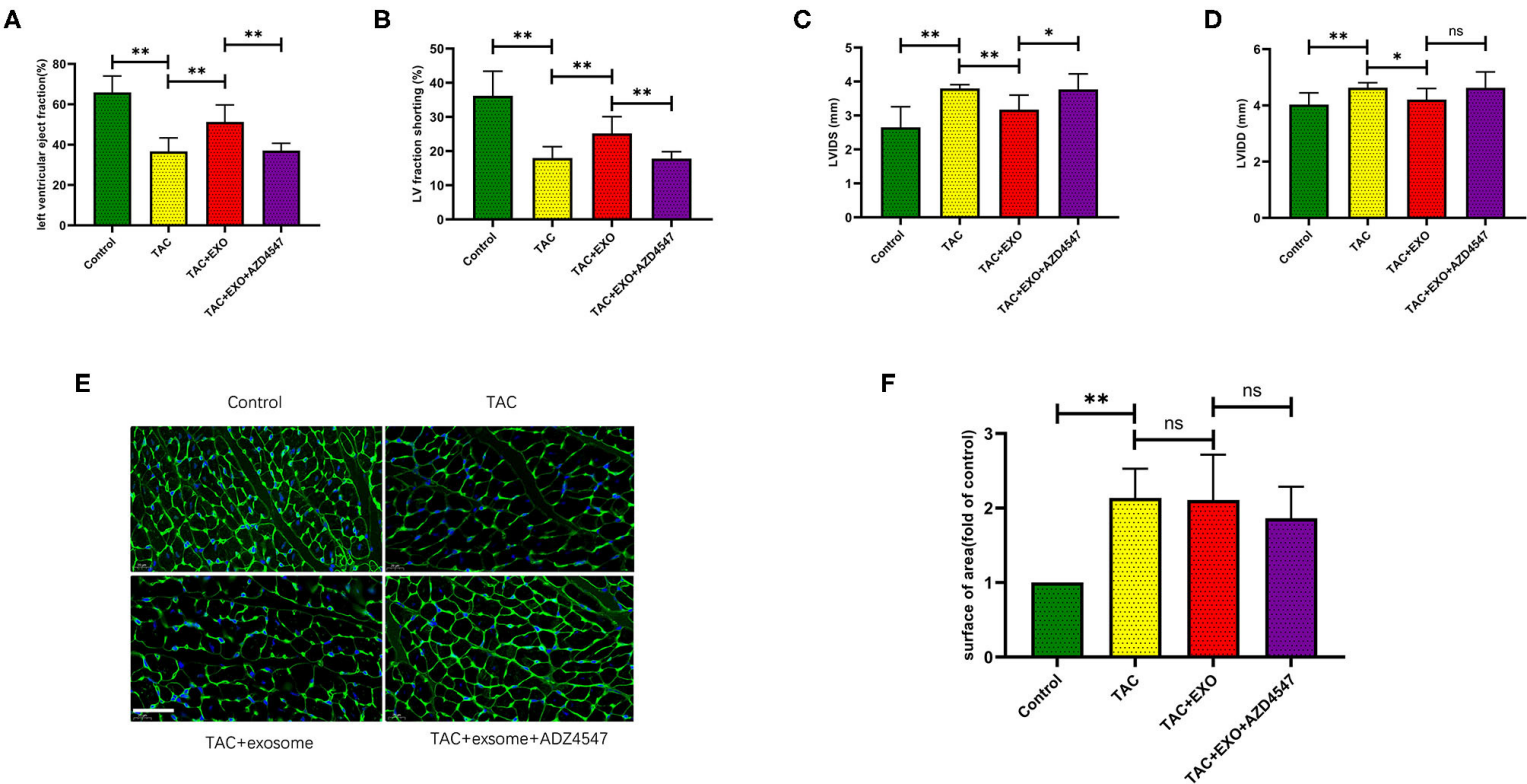
ESCs-derived exosomes effectively rescued heart failure after TAC. **(A-D)** Exosomes treatment significantly rescued the heart function after TAC which was attenuated by AZD4547 administration. **(E,F)** TAC induced myocardium cell cross section area increasement significantly while exosomes treatment had no effect on cell size. (*n* = 5-8, scale bar, 100 um). *p < 0.05, **p < 0.01.

Compared to PBS, exosome treatment significantly rescued LVEF and Fraction shorting (FS) (*p* < 0.01) in mice after TAC (Figures 3A,B). Both LVIDD (*p* < 0.05) and LVIDS (*p* < 0.01) were significantly reduced in the exosome-treated mice group relative to the control group (Figures 3C,D). All the above effects were attenuated by AZD4547 administration (Figures 3A–D).

### ESC-Derived Exosomes Had No Effect on Cardiac Fibrosis and Myocardium Cell Cross Section Area

**Figure 4:**
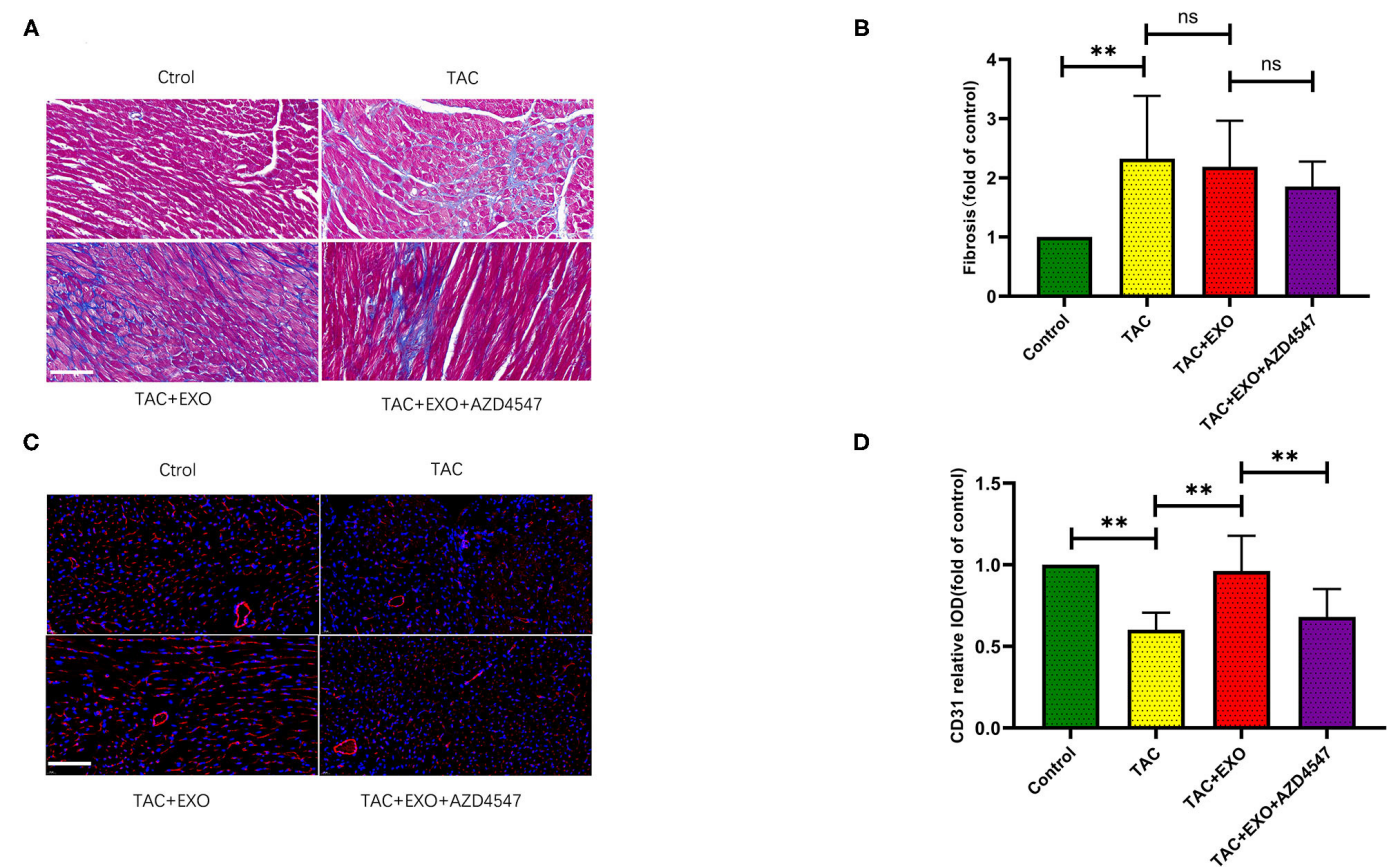
ESCs-derived exosomes increased cardiac angiogenesis and have no effect on the interstitial fibrosis. **(A,B)** TAC induced marked myocardial fibrosis, as evidenced by Masson staining. However, exosomes administration had no significant effect on the TAC induced myocardial fibrosis (scale bar, 100 um). **(C,D)** CD31 expression was markedly decreased in the TAC group which could be obviously rescued by exosomes administration (*n* = 5–8). *p < 0.05,**p < 0.01.

TAC induced increased cell area (Figures 3E,F, *p* < 0.01) and marked myocardial fibrosis (Figures 4A,B, *p* < 0.01) as shown by WGA and Masson staining. Exosome administration had no significant effect on TAC-induced myocardial fibrosis and cell area enlargement.

### ESC-Derived Exosomes Alleviated Cardiac Microvessel Impairment in TAC Mice Which Can Be Attenuated by AZD4547 Administration

Microvessel density was markedly decreased in the TAC group relative to the control group which could be obviously rescued by exosome administration. This effect was significantly attenuated by AZD4547 administration (Figures 4C,D, *p* < 0.01). These findings indicated that ESC-derived exosome administration can promote myocardial angiogenesis and mitigate the reduction in micro-vessel density induced by TAC in a FGF2 dependently way.

## Discussion

Here, we provide compelling evidence that (1) ESC-derived exosomes significantly attenuate TAC-induced heart failure by promoting myocardial angiogenesis and (2) FGF2 signaling plays vital roles in the myocardial angiogenesis induced by ESC-derived exosomes.

Our study revealed that FGF2 was highly enriched in ESC-derived exosomes. FGF2 has been confirmed to stimulate the proliferation of mesenchymal cells such as fibroblasts, endothelial cells, and smooth muscle cells (14). Endogenous FGF2 has a significant cardioprotective effect against ischemia-reperfusion injury (15). Deletion of FGF2 has been shown to result in decreased endothelial proliferation and vascular density in the infarcted myocardium of mice (16). FGFR1 and FGFR2 DKO mice, which exhibit endothelial cell-specific disruption of FGF2 function, have been shown to have significantly worsened cardiac function than controls after ischemia-reperfusion injury as well as significantly decreased vessel density (17). Our study provides the first demonstration that the administration of ESC-derived exosomes containing a high abundance of FGF2 can promote myocardial angiogenesis after aortic banding.

As ESCs-derived exosomes contain many proteins and microRNAs in addition to FGF2, we performed *in vitro* experiments to investigate the role of FGF2 in the angiogenesis induced by ESCs-derived exosomes. We found that ESC-derived exosomes can significantly increase tube formation and that this effect could be largely mitigated by FGF2R siRNA. All the above findings suggested that FGF2-FGF2R signaling played a vital role in the myocardial angiogenesis induced by ESC-derived exosomes.

FGF2 stimulates fibroblast and matrix production (16). Schultz and colleagues reported that FGF2-KO mice exhibited reduced interstitial fibrosis after aortic banding (18). However, in our study, administration of exosomes containing abundant FGF2 did not increase myocardial interstitial fibrosis. Fibroblast growth factor 2 (FGF-2) can be categorized as high molecular weight (20 kDa) or low molecular weight (18 kDa) which exert distinct biological activities: low molecular weight FGF-2 promoted sustained cardioprotection and angiogenesis, while high molecular weight FGF-2 promoted myocardial hypertrophy and reduced contractile function (19). Our Proteomic analysis showed that ESC-derived exosomes contained rich of low molecular weight (18 kDa) FGF2 instead of high molecular weight (20 kDa). This may explain the absent of increased myocardial interstitial fibrosis in the exosome treated group in our study. Another possible explanation was that other signaling molecules than FGF2 in exosomes may also be involved in the ventricular remodeling process and offset FGF2-induced interstitial fibrosis.

## Conclusions

ESC-derived exosomes attenuated TAC-induced heart failure by promoting myocardial angiogenesis. FGF2 signaling may played vital roles in the myocardial angiogenesis induced by ESC-derived exosomes.

## Data Availability Statement

The datasets presented in this study can be found in online repositories. The names of the repository/repositories and accession number(s) can be found below: ProteomeXchange, accession no: PXD015449.

## Ethics Statement

The animal study was reviewed and approved by Ethics Committee of the First Affiliated Hospital of Soochow University.

## Author Contributions

XL and ZW designed the study. YP, MM, and DW wrote the manuscript and performed experiments. XW and JX analyzed these data. ZW, LH, and XL revised the article. All authors have read and approved the final version of the manuscript, and, therefore, have full access to all the data in the study and take responsibility for the integrity and security of the data.

## Conflict of Interest

The authors declare that the research was conducted in the absence of any commercial or financial relationships that could be construed as a potential conflict of interest.
