# Supplementary figures and images for: Embryonic Stem Cell-Derived Exosomes Attenuate Transverse Aortic Constriction Induced Heart Failure by Increasing Angiogenesis
Source: Front Cardiovasc Med. 2021 Jun 28;8:638771. doi: 10.3389/fcvm.2021.638771 (PMC8273241; doi:10.3389/fcvm.2021.638771)

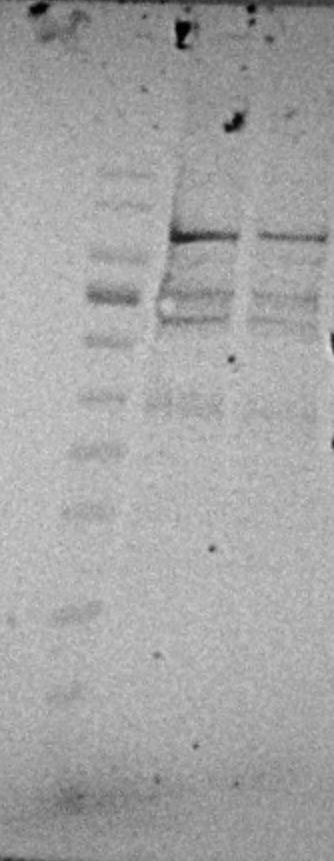

Supplement: Supplementary file 5 [file Image_1.JPEG]

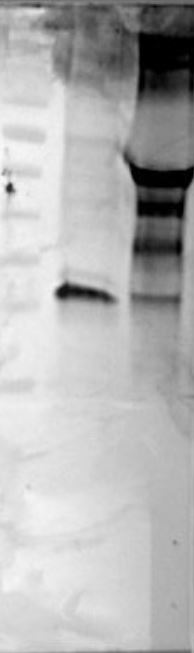

Supplement: Supplementary file 6 [file Image_2.JPEG]
